# Monoclonal antibodies targeting the calcitonin gene-related peptide pathway improve the effectiveness of acute medication—a real-world study

**DOI:** 10.1007/s10072-024-07380-4

**Published:** 2024-02-10

**Authors:** Chiara Rosignoli, Valeria Caponnetto, Agnese Onofri, Vittorio Trozzi, Lorenzo Tartaglione, Marcello Silvestro, Antonio Russo, Simona Sacco, Raffaele Ornello

**Affiliations:** 1 Department of Biotechnological and Applied Clinical Sciences, University of L’Aquila, L’Aquila, Italy; 2 Department of Life, Health and Environmental Science, University of L’Aquila, L’Aquila, Italy; 3 Department of Advanced Medical and Surgical Sciences, University of Campania “Luigi Vanvitelli”, 80138 Naples, Italy

**Keywords:** Migraine, Calcitonin gene-related peptide, Migraine treatment, Acute medication

## Abstract

**Background:**

One of the aims of migraine prevention is to improve response to acute migraine treatments. The aim of the present study was to assess whether monoclonal antibodies targeting the CGRP pathway (CGRP-mAbs) can improve the perceived efficacy of acute treatments.

**Methods:**

We included and followed up patients with chronic or episodic migraine from the Headache Centers of Avezzano-L’Aquila and Naples treated with CGRP-mAbs from March 2021 to December 2022. All patients filled out the Migraine Treatment Optimization Questionnaire (MTOQ), the Headache Impact Test (HIT-6), and the Migraine Impact and Disability Assessment Scale (MIDAS) at baseline and 3–6 months after the start of treatment with CGRP-mAbs.

**Results:**

Sixty-five patients (81.3%) completed the 6-month follow-up. Most patients were female (55, 84.6%), with a median age of 46 years (IQR 39–56). Median MTOQ score increased from 8 (interquartile range [IQR] 4–13) at baseline to 15 (IQR 11–17) at 3 months (*p* < 0.001) and 16 (IQR 13–17) at the 6-month follow-up (*p* < 0.001). Median migraine days over 90-day periods decreased from 40 (IQR 24–60) to 24 (IQR 15–30) at 3 months (*p* < 0.001) and to 20 (IQR 12–24) at 6 months (*p* < 0.001). Median monthly intake of acute medication decreased from 55 doses (IQR 29–80.5) to 24 doses (IQR 15–40) at 3 months and 18 doses (IQR 11–30) at 6 months (*p* < 0.001).

**Conclusions:**

We showed that 6 months of preventive treatment with CGRP-mAbs led to a significantly better effectiveness of acute treatments, paralleled by decreased monthly migraine days and acute treatment intake.

**Supplementary Information:**

The online version contains supplementary material available at 10.1007/s10072-024-07380-4.

## Introduction

Migraine, ranked as the third most prevalent disorder globally, constitutes the leading cause of disability among individuals under 50 years of age [1]. Migraine treatment encompasses acute medications to address each single attack and preventive medications aimed at reducing both the frequency and severity of episodes. The interplay between these acute and preventive modalities is pivotal, as preventive treatments can augment the efficacy of acute medication [2–6]. Despite their significance, there is a paucity of systematic assessments regarding the extent to which preventive medications enhance the effectiveness of acute treatments.

Monoclonal antibodies acting on the calcitonin gene-related peptide (CGRP) pathway (CGRP-mAbs) represent the first preventive agents specifically designed for migraine [7, 8]. The efficacy parameters evaluated in randomized controlled trials and real-world studies on CGRP-mAbs predominantly encompass reductions in headache frequency, intensity, and acute medication consumption [9–13]. However, emerging evidence suggests that CGRP-mAbs exert a broader influence on patients’ quality of life, ameliorating symptoms of anxiety, depression, and pain catastrophizing, while also reducing the number of migraine days [14, 15]. In clinical practice, it would be important to investigate whether CGRP-mAbs increase the efficacy of acute treatments; knowledge of this aspect can significantly inform therapeutic discussions between healthcare professionals and patients. The aim of the present study was to investigate whether anti-CGRP monoclonal antibodies can enhance acute treatment responses and patient-perceived efficacy of these treatments within a real-world context.

## Materials and methods

### Study design

We conducted a prospective, multicenter, observational study involving consecutive migraine patients who sought treatment at the two tertiary headache centers of Avezzano-L’Aquila and Naples. Both centers have extensive expertise in the administration and management of advanced migraine treatments. These centers routinely employ specific paper diaries to monitor headache frequency, intensity, and the utilization of acute medications among their patients.

### Inclusion and exclusion criteria

We adhered to the following inclusion criteria:

- Age ≥ 18 years
- Male or female sex
- Diagnosis of migraine with or without aura according to the International Classification of Headache Disorders (ICHD-3 [16])
- Meeting the criteria for prescription of CGRP-mAbs, i.e., ≥ 8 debilitating monthly headache days, failure of ≥ 3 preventive medication classes due to inefficacy, poor tolerability, or contraindication, and Migraine Impact and Disability Assessment Scale (MIDAS) score ≥ 11
- Provided informed consent for the study

Patients with chronic migraine and medication overuse headache were considered for inclusion, provided that they met the diagnostic criteria for migraine. Patients concurrently using preventive medications for migraine were eligible for inclusion if they had maintained a consistent dosage for at least 90 days before initiating treatment with CGRP-mAbs and continued this regimen throughout the study observation period. Concurrent treatment with onabotulinumtoxinA was not allowed by the rules of reimbursement for CGRP-mAbs issued by the Italian Medicine Agency (AIFA). All patients included in the present study were treated in a reimbursement regime.

Patients who failed to meet the inclusion criteria and those who had previously received anti-CGRP treatments prior to their initial study visit were excluded from the study.

### Ethical procedures

All ethical procedures necessary to ensure the protection of the rights and welfare of participants were strictly adhered to. This study was approved by the Internal Review Board of the University of L’Aquila with protocol number 10/2021.

Prior to participating in the study, each participant was adequately informed of the details of the study, including the aims, risks, and potential benefits. An informed consent was provided to each participant, clearly describing the terms and conditions of their participation. Participants were given the opportunity to ask questions and signed the informed consent voluntarily, thus confirming their informed participation in the study.

### Study procedures

Patient recruitment for this study spanned a 12-month period, starting in July 2021 and concluding in June 2022. Subsequently, a 6-month follow-up phase that extended until December 2022 was implemented.

The study protocol encompassed three key evaluation points: a baseline visit and two follow-up visits. During the baseline visit, we recorded biographical information, including migraine duration (in years), prior preventive treatment failures, presence of medication overuse (MO), and the number of migraine days and acute medication intakes within the preceding 90 days. In accordance with the established clinical practices at the participating centers, we assessed the degree of disability and the impact of migraine on daily activities by administering the Italian version of the Headache Impact Test (HIT-6) and the Migraine Impact and Disability Assessment Scale (MIDAS). After the baseline visit, patients were prescribed erenumab, fremanezumab, or galcanezumab at the discretion of the treating physician. To track migraine frequency and the usage of acute medication, patients maintained paper-based headache diaries. After 3 and 6 months, patients underwent two follow-up visits, wherein their headache diaries were reviewed alongside assessments of MIDAS, HIT-6, and MTOQ. To ensure consistency in assessments across the various CGRP-mAbs (with erenumab administered every 28 days and the other mAbs every 30 days), data from the preceding 90 days were employed to evaluate both the 3-month and 6-month outcomes.

### Evaluation of improvement in response to acute treatment

In addition to these standard assessments, patients were requested to complete the Migraine Treatment Optimization Questionnaire (MTOQ), a tool designed for evaluating patient satisfaction with acute treatment, medication tolerability, and the incidence of adverse events [17]. Validated in multiple languages, including English, French, German, Spanish, and Italian, the original version of the MTOQ comprises 19 questions, each with a “Yes” or “No” responses (Supplemental Fig. 1). A cumulative score, ranging from 0 to 19, is computing by attributing one point for each affirmative response, with the option to assess subgroups using the MTOQ-15 (15 questions) or MTOQ-5 (five questions) variants. The MTOQ assesses, after acute migraine treatment, return to normal function, absence of pain at 2 h, prolonged pain relief at 24 h, tolerability, comfort in making plans, and perceived control. To give a complete account of the response to acute medication, we recorded the results of the MTOQ-19 instead of shorter versions of the questionnaire.

### Outcomes

The primary outcome of the study was the change in MTOQ score from baseline to 3 and 6 months. Secondary outcomes included the change in monthly headache days, monthly migraine days, and acute medication intakes (the number of doses of acute medications taken in 1 month) from baseline to 3 months and 6 months.

We also performed a subgroup analysis in patients with MO to assess differences in MTOQ scores and medication intake over the 6-month observation period.

### Statistical analysis

The study included all patients who remained in follow-up for the entire 6-month duration, irrespective of treatment discontinuation due to ineffectiveness, adverse events, or patient preference. In cases where patients discontinued treatment for any reason, outcomes were reported utilizing a “last observation carried forward” approach. Patients who withdrew consent or were lost to follow-up were excluded from the analyses.

To assess whether the improvement in the effectiveness of acute medication was independent from the effectiveness of mAbs, we tested the correlation between the change in MTOQ score and each of the secondary outcomes. All tests were performed at 3 months and at 6 months to ascertain consistency over different time points. To assess the role of MO in the response to acute medication, we performed subgroup analyses for each outcome for patients with and without MO.

Patient characteristics and sociodemographic information were summarized using either numbers and percentages or medians and interquartile ranges (IQRs), as appropriate. The assessment of changes in continuous parameters, including the number of headache and migraine days and analgesic intake, was conducted through non-parametric tests, comparing baseline data with follow-up measurements. Specifically, the Wilcoxon signed-rank test was employed to determine the significance of differences between medians at baseline and after the 6-month follow-up. Additionally, Spearman’s correlation analysis was utilized to explore relationships between outcome variables. A statistical significance threshold was set at *p* < 0.05. We chose non-parametric tests to maintain conservative estimates.

The MTOQ has never been used as a parameter for evaluating the effectiveness of preventive treatments for migraine, so there are no studies available in the literature that allow the sample to be calculated. It is estimated, however, that the inclusion of 34 patients completing the study allows for a mean effect (Rho = 0.5) on the primary outcome with a 95% confidence interval and a statistical power of 90%.

## Results

### Patients’ characteristics

**Fig. 1:**
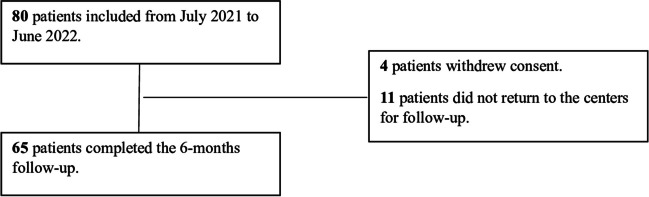
Flowchart of patients’ inclusion

Of the initial cohort of 80 patients, 4 patients withdrew consent for the study and 11 did not return to the centers for follow-up; 65 (81.3%) were included in the final analysis. No patient discontinued treatment due to ineffectiveness or any other reason within the follow-up period (Fig. 1). Table 1 shows the patients’ characteristics. Most patients were female (55, 84.6%) or had a diagnosis of chronic migraine (48, 75.0%). Thirteen patients (20.3%) experienced migraine with aura. The median age of the 65 patients was 46 years (IQR 39–56). The median duration of migraine was 26 years (IQR 20–36), and patients reported a median of 3 (IQR 1–3) previous failures of preventive treatments before starting anti-CGRP monoclonal antibodies. Specifically, 16 patients (24.6%) received erenumab 140 mg/month, 30 patients (47.7%) received fremanezumab 225 mg/month, and 19 patients (27.7%) received galcanezumab 120 mg/month (with 240-mg loading dose). None of the patients received quarterly administrations of fremanezumab.

**Table 1:** Characteristics of the study population. *IQR* interquartile range

Female, *n* (%)	55 (84.6)
Age, median (IQR)	46 (39–56)
Chronic migraine, *n* (%)	48 (75.0)
Medication overuse, *n* (%)	30 (47.7)
Aura, *n* (%)	13 (20.3)
Migraine duration, median (IQR)	26 (20–35.5)
Failures in preventive treatment, median (IQR)	3 (1–3)
Erenumab, *n* (%)	16 (24.6)
Fremanezumab, *n* (%)	30 (47.7)
Galcanezumab, *n* (%)	19 (27.7)

### Primary outcome

**Fig. 2:**
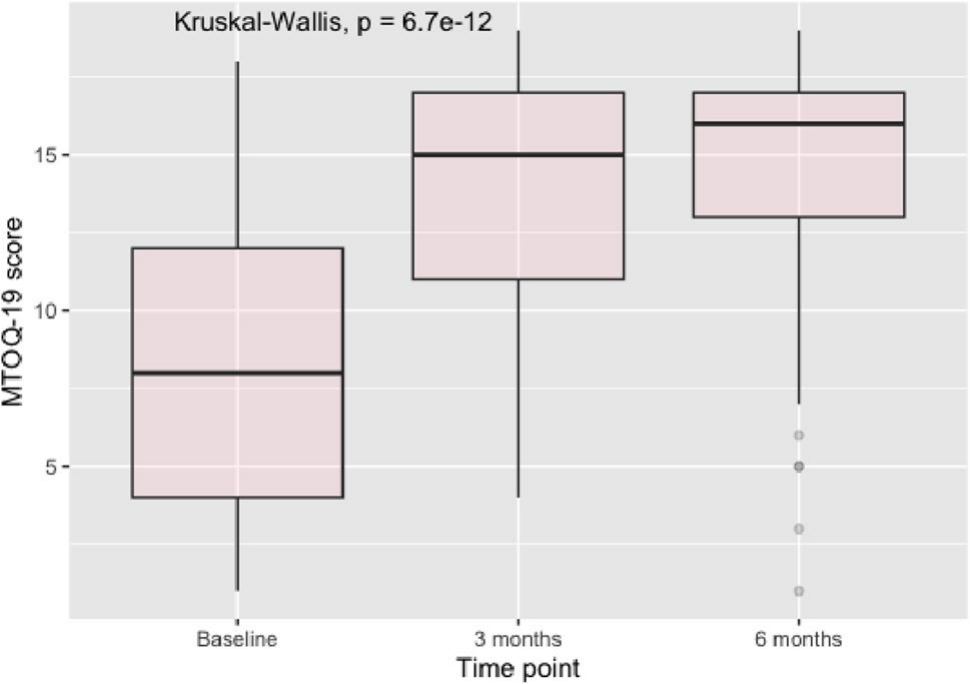
Box plot of change in Migraine Treatment Optimization Questionnaire-19 in the 65 patients with a 6-month follow-up. All changes from baseline to 3 months and 6 months have a *p* < 0.001 (Wilcoxon test)

Median MTOQ scores increased from 8 (IQR 4–13) at baseline to 15 (IQR 11–17) at 3 months and 16 (IQR 13–17) at the 6-month follow-up (*p* < 0.001; Fig. 2), indicating an improvement in the perceived effectiveness of acute medication from baseline.

A significant improvement was also observed in the two subscales of the MTOQ: scores in MTOQ15 improved significantly from 5 (IQR 3–9) at baseline to 11 (IQR 9–13) at 3 months and 13 (IQR 10–13) at 6 months (*p* < 0.001). The MTOQ5 scores increase from 2 (IQR 1–4) at baseline to 4 (IQR 3–5) at 3 months and to 5 (IQR 4–5) at 6 months (*p* < 0.001).

### Secondary outcomes

**Fig. 3:**
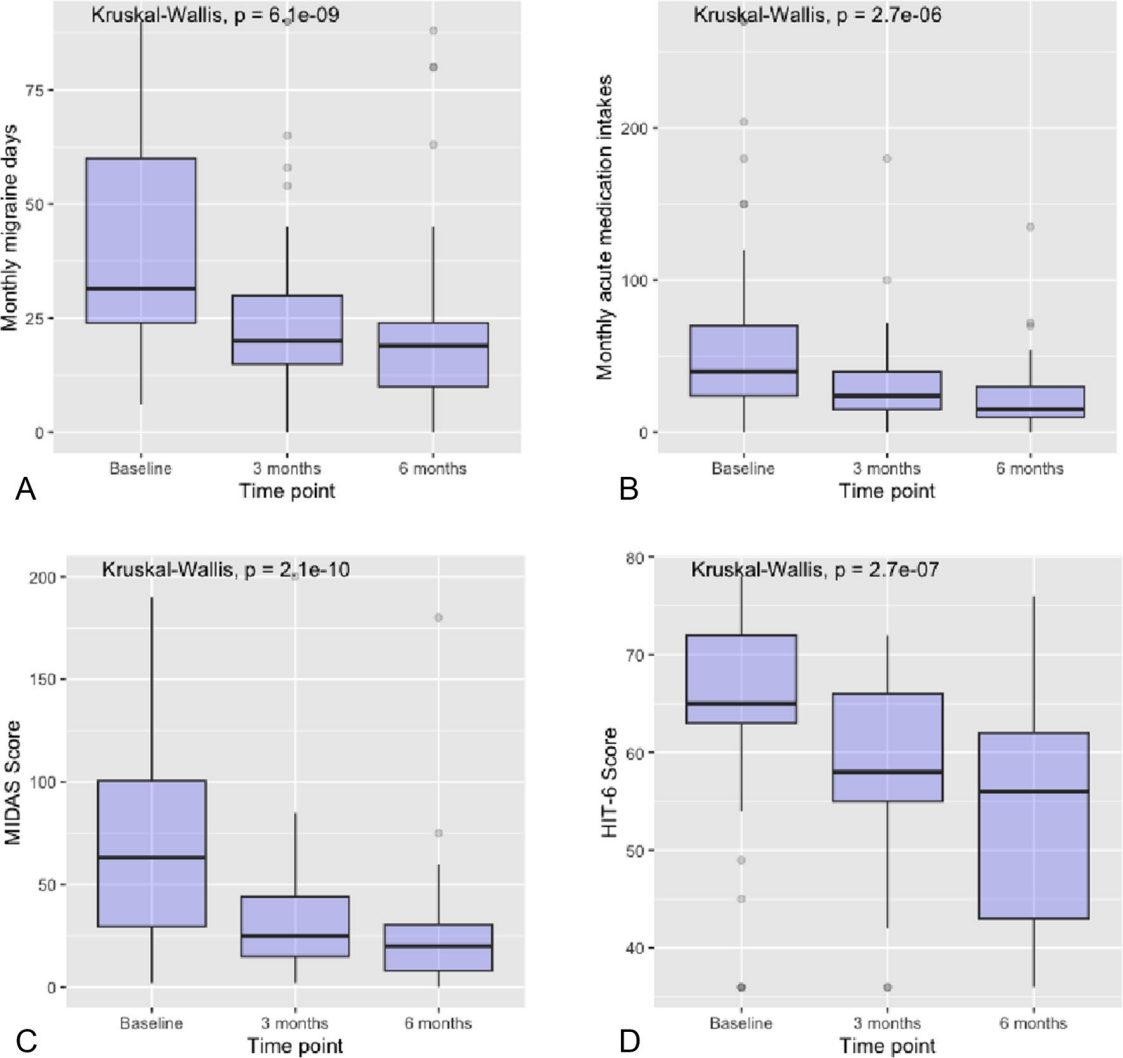
Box plots of monthly migraine days (**A**), medication intake (**B**), Migraine Impact and Disability Assessment Scale (**C**), and Headache Impact Test-6 scores (**D**) at baseline, 3 months, and 6 months. All comparisons have *p* < 0.001 compared with baseline

Regarding the efficacy parameters, median migraine days over 90-day periods decreased significantly from a baseline median of 40 (IQR 24–60) to 24 (IQR 15–30) at 3 months and to 20 (IQR 12–24) at 6 months (*p* < 0.001 at each time point compared to baseline; Fig. 3A). Patients initially reported a median of 55 intakes (IQR 29–80.5) of acute medications (NSAIDs, opioids, triptans) at baseline, which subsequently decreased to 24 intakes (IQR 15–40) at 3 months and 18 intakes (IQR 11–30) at 6 months (*p* < 0.001; Fig. 3B).

Concurrently, the median MIDAS score decreased from 68 (IQR 45–105) at baseline to 25 (IQR 15–44) at 3 months and 20 (IQR 10–32) at 6 months (*p* < 0.001 at each time point compared to baseline Fig. 3C). The median HIT-6 score declined from 66 (IQR 62–72) to 59 (IQR 56–66) at 3 months and 56 (IQR 43–66) at 6 months (*p* < 0.001; Fig. 3D).

**Fig. 4:**
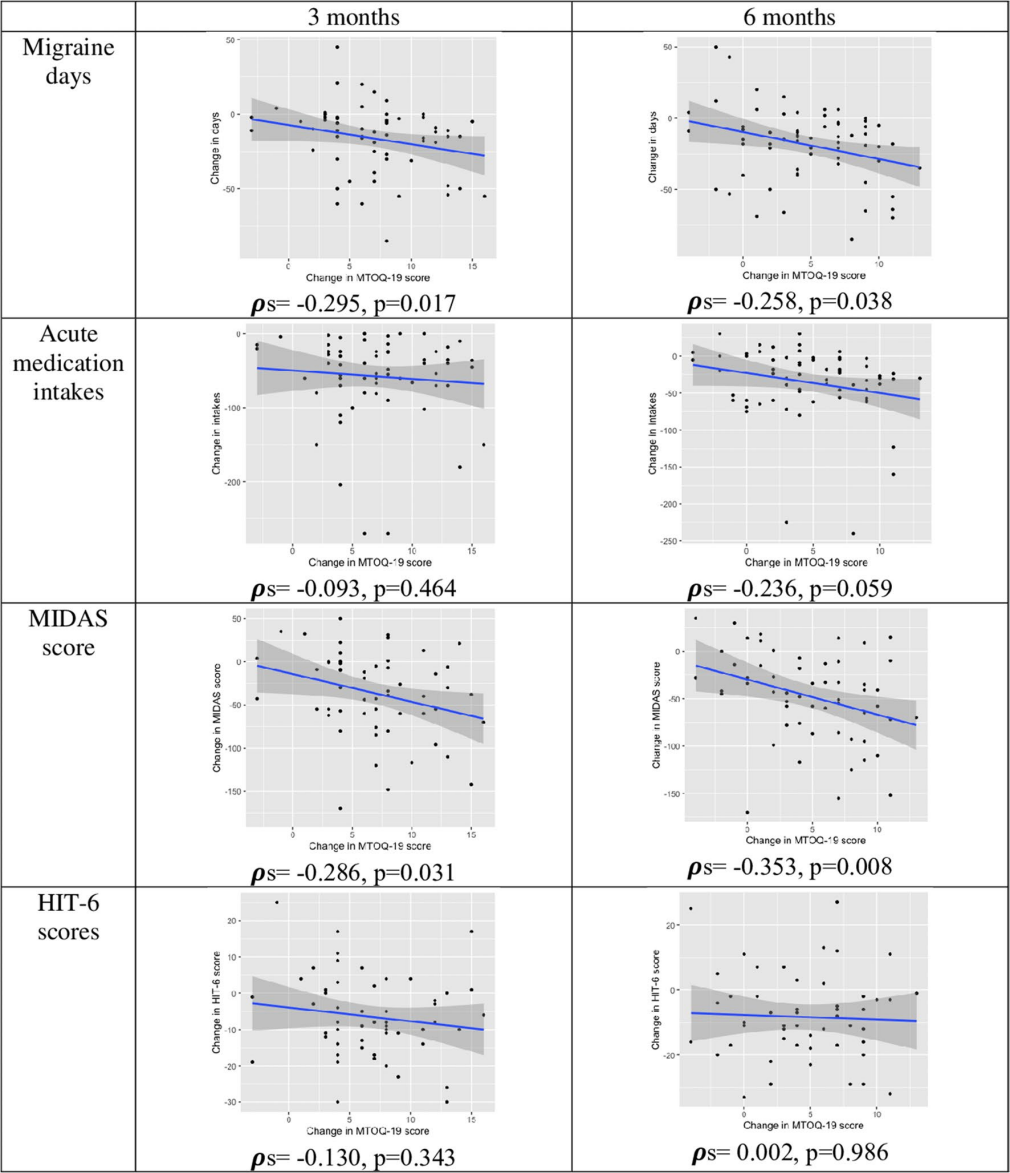
Correlations between changes in Migraine Treatment Optimization Questionnaire scores and changes in migraine days, acute medication intakes, Migraine Impact and Disability Assessment Scale, and Headache Impact Test-6 scores at 3 months and 6 months. *HIT-6* Headache Impact Test-6, *MIDAS* Migraine Impact and Disability Assessment Scale

We found a negative correlation between increased MTOQ scores and decreased migraine days at 3 months (ρs =  − 0.295, *p* = 0.017) and at 6 months (ρs =  − 0.258, *p* = 0.038), suggesting a relationship between the improved perceived effectiveness of acute medication and the decrease in migraine frequency. Conversely, the increase in MTOQ scores did not exhibit any significant correlation with decreased acute medication intakes either at 3 months (ρs =  − 0.093, *p* = 0.464) or at 6 months (ρs =  − 0.58, *p* = 0.647), which suggests that the improved perceived effectiveness of acute medication was independent of the change in medication intake. We found a correlation between the increase in MTOQ score and the decrease in MIDAS score (3 months: ρs =  − 0.286, *p* = 0.031; ρs =  − 0.353 *p* = 0.008), which is influenced by migraine frequency. Conversely, we found no correlation between the increase in MTOQ scores and the decrease in HIT-6 scores (3 months: ρs =  − 0.130, *p* = 0.343; 6 months: ρs =  − 0.353 *p* = 0.008). Correlations are graphically reported in Fig. 4.

### Subgroup analyses according to the presence of medication overuse

Subgroup analyses performed according to the presence of MO at baseline showed that the decrease in MMDs, acute medication intakes, MIDAS, and HIT-6 scores and the increase in MTOQ scores were comparable in patients with and without MO at baseline (Supplemental Fig. 2).

## Discussion

In our study, we demonstrated that the use of CGRP mAbs in the preventive treatment of migraine is associated with a significant increase in the perceived efficacy of acute medication as documented by a reliable and validated instrument. We found a similar effect of treatment in patients with and without medication overuse. This is a finding of particular relevance for migraine patients, as the efficacy of acute treatment is critical for relief of painful and disabling symptoms. One of the fundamental goals of migraine prevention is to improve the response to acute treatment. The assessment of patient satisfaction with acute treatment is of particular importance in clinical practice because of its strong association with migraine-related disability [18–20]. However, this objective remains underexplored, even in the context of randomized controlled trials and real-world studies examining CGRP-mAbs, which focused more on acute medication intake than on their effectiveness [3, 4, 21, 22]. To address this, the Migraine Treatment Optimization Questionnaire (MTOQ) was developed and validated, offering a means to quantify acute medication response. We chose to utilize the MTOQ-19 score, in comparison with the abbreviated MTOQ-4, with the intention of precisely measuring the change in patients’ response to acute medication in relation to their response to CGRP-mAbs. The MTOQ investigates not only the effectiveness of the drug in terms of eliminating pain, but also assesses the patient’s ability to return quickly to his normal activities (work, family, leisure, or social activities). The switch from frequent and often ineffective acute treatments to more effective symptom relief medication might not only relieve physical pain but also offer considerable psychological relief given the prospect of a better quality of life, with fewer interruptions due to migraine attacks.

When considering the relationship between the improved effectiveness of acute medication and other components of response to CGRP-mAbs, we obtained two significant findings. First, the improved response to acute medication, as indicated by higher MTOQ-19 scores, correlated with the decrease in migraine frequency. A possible explanation for this finding lies in desensitization. The efficacy of acute migraine medication may diminish with repeated use, potentially contributing to central sensitization to pain [23, 24]. Conversely, effective migraine prevention might increase the effectiveness of acute medication by mitigating central sensitization. The second finding of our study was that the improved response to acute medication did not correlate with the decrease in medication intake. This finding suggests that in real-world settings, the improvement in acute medication response occurs independently of a reduction in the consumption of acute medications. This discrepancy can be attributed to non-pharmacological factors. Both acute and preventive migraine medications are susceptible to placebo and nocebo effects [25]. The treatment outcomes for any patient may be bolstered by effective migraine prevention and the supportive environment of headache centers.

Another explanation could be the presence of MO in almost half of the sample and the strong behavioral component associated with the conditions. In fact, we observed a lower reduction in drug intake, despite a higher perceived benefit of acute treatment. These patients may be reluctant to change their acute medication pattern and may be anxious or worried if a dose is missed. As shown in previous studies, MOH patients tend to develop a dependence from substances because of the need to cope with recurrent pain.

To our knowledge, our study is the first real-world investigation dedicated to elucidating enhancements in acute migraine medication response associated with CGRP-mAb treatment. These parameters bear clinical relevance, and our findings could be important in discussing with patients in clinical practice. Nevertheless, our study has some limitations. First and foremost, it relied on a relatively limited patient cohort recruited within a finite timeframe. Additionally, the follow-up period of up to 6 months may have been insufficient to detect significant alterations in the migraine biology, particularly in patients with long-standing histories of chronic migraine and/or medication overuse. Variability in our findings may have arisen from the inclusion of patients treated with all clinically available CGRP-mAbs. Furthermore, our assessment relied solely on clinical judgments and patient self-reports, without objective measurements of sensitization or analgesic effects of medications. Consequently, we could not adequately distinguish between non-pharmacological effects and pharmacological effects. Finally, it was not possible to perform sub-analyses according to the type of acute treatment taken, as most patients were on non-steroidal anti-inflammatory drugs.

## Conclusion

Our study shows a significant increase in the effectiveness of acute treatment following the intake of CGRP-mAbs. Therefore, we demonstrated that migraine preventive medication, and mostly migraine-specific drugs, can meet the goal of improving response to acute treatments.

### Supplementary Information

Below is the link to the electronic supplementary material.

Supplementary file1 (DOCX 397 KB)
