# Supplementary material for: Monoclonal antibodies targeting the calcitonin gene-related peptide pathway improve the effectiveness of acute medication—a real-world study
Source: Neurol Sci. 2024 Feb 10;45(7):3305–12. doi: 10.1007/s10072-024-07380-4 (PMC11176241; doi:10.1007/s10072-024-07380-4)

Supplemental Figure 1. MTOQ-19 Questionnaire English (a) and Italian (b) versions.

**A** Migraine Treatment Optimization Questionnaire – English version

|  | **Yes** | **No** |
| --- | --- | --- |
| 1. Are you able to function normally within 2 hours after taking your migraine medication? |  |  |
| 2. Are you able to resume your normal activities (ie, work, family, leisure, and social activities) within 2 hours after taking your migraine medication? |  |  |
| 3. Are you able to quickly return to your normal activities (ie, work, family, leisure, social activities) after taking your migraine medication? |  |  |
| 4. Do any feelings of tiredness, irritability, sadness, anger, and anxiety disappear within 2 hours after taking your migraine medication? |  |  |
| 5. After taking your migraine medication does your migraine headache disappear within 2 hours for most attacks? |  |  |
| 6. After taking your migraine medication are you pain free within 2 hours for most attacks? |  |  |
| 7. After taking your migraine medication do you get significant relief of your migraine headache within 2 hours for most attacks? |  |  |
| 8. Can you count on your migraine medication to relieve your pain within 2 hours for most attacks? |  |  |
| 9. Does your migraine medication work consistently in the majority of your attacks? |  |  |
| 10. Does your migraine medication usually relieve the symptoms which accompany your migraine headache (ie, nausea, sensitivity to light or to sound)? |  |  |
| 11. Does your migraine medication relieve the pain and the symptoms which accompany your migraine headache (ie, nausea, sensitivity to light or sound) within 2 hours, most of the time? |  |  |
| 12. Does one dose of your migraine medication relieve your migraine headaches and keep them away for at least 24 hours, for most attacks? |  |  |
| 13. When you have a migraine headache, do you need to take your migraine medication only once to treat the attack? |  |  |
| 14. Does one dose of your migraine medication usually relieve your headache and keep it away for at least 24 hours? |  |  |
| 15. With your current migraine medication, do you have side effects that disturb you? |  |  |
| 16. Is your migraine medication well tolerated? |  |  |
| 17. Do you have troublesome side effects after using your current migraine medication? |  |  |
| 18. Are you comfortable enough with your migraine medication to be able to plan your daily activities? |  |  |
| 19. After taking your migraine medication do you feel in control of your migraines enough so that you feel there will be no disruption to your daily activities? |  |  |

**B** Migraine Treatment Optimization Questionnaire – Italian version (I-MTOQ)

|  | **Si** | **No** |
| --- | --- | --- |
| 1. Riesci a tornare alla normalità delle tue funzioni entro 2 ore dall'assunzione del farmaco per l'attacco di emicrania? |  |  |
| 2. Riesci a riprendere le tue attività quotidiane (lavorative, familiari, di svago, sociali) entro 2 ore dall’assunzione del farmaco per l’attacco di emicrania? |  |  |
| 3. Riesci a ritornare rapidamente alle tue attività normali (lavorative, familiari, di svago, sociali) dopo aver assunto il farmaco per l’attacco di emicrania? |  |  |
| 4. Eventuali sensazioni di stanchezza, irritabilità, tristezza, rabbia e ansia spariscono entro 2 ore dall’assunzione del farmaco per l’attacco di emicrania? |  |  |
| 5. Dopo aver assunto il farmaco per l’attacco di emicrania, il tuo mal di testa da emicrania sparisce entro 2 ore nella maggior parte degli attacchi? |  |  |
| 6. Dopo aver assunto il farmaco per l’attacco di emicrania, sei libero dal dolore entro 2 ore nella maggior parte degli attacchi? |  |  |
| 7. Dopo aver assunto il farmaco per l’attacco di emicrania, ricavi un significativo sollievo dal tuo mal di testa da emicrania entro 2 ore nella maggior parte degli attacchi? |  |  |
| 8. Puoi contare sul tuo farmaco per l’attacco di emicrania per alleviare il dolore entro 2 ore nella maggior parte degli attacchi? |  |  |
| 9. Il tuo farmaco per l’attacco di emicrania funziona costantemente nella maggior parte degli attacchi? |  |  |
| 10. Il tuo farmaco per l’attacco di emicrania allevia, di solito, i sintomi che accompagnano il mal di testa da emicrania (nausea, fastidio per le luci o 1 suoni)? |  |  |
| 11. Il tuo farmaco per l’attacco di emicrania allevia il dolore e i sintomi che accompagnano il mal di testa (nausea, fastidio per le luci o i suoni) entro 2 ore nella maggior parte dei casi? |  |  |
| 12. Una sola dose di farmaco per l'attacco di emicrania riesce ad alleviare i tuoi mal di testa da emicrania e tenerli lontani per almeno 24 ore, nella maggior parte degli attacchi? |  |  |
| 13. Quando hai mal di testa da emicrania, ti basta assumere il tuo farmaco antiemicranico solo una volta per trattare l'attacco? |  |  |
| 14. Una sola dose del tuo farmaco per l'attacco di emicrania, di solito, allevia i tuoi mal di testa e li tiene lontani per almeno 24 ore? |  |  |
| 15. Il farmaco per l'attacco di emicrania che stai utilizzando ti dà effetti collaterali che ti disturbano? |  |  |
| 16. Il tuo farmaco per l'attacco di emicrania è ben tollerato? |  |  |
| 17. Hai effetti collaterali fastidiosi dopo aver assunto il farmaco per l'attacco di emicrania che usi attualmente? |  |  |
| 18. Sei abbastanza a tuo agio con il farmaco per l'attacco di emicrania da poter programmare le tue attività quotidiane? |  |  |
| 19. Dopo aver assunto il tuo farmaco per l'attacco di emicrania, senti di poter controllare la tua emicrania tanto da pensare che non ci sarà alcuna interruzione nelle tue attività quotidiane? |  |  |

Supplemental Figure 2. Box plots of monthly migraine days (a), medication intake (b), Migraine Impact and Disability Assessment Scale (c) and Headache Impact Test-6 scores (d) at baseline, 3 months, and 6 months according to the presence of medication overuse (grey boxes= absent; blue boxes= present). All comparisons have p<0.001 compared with baseline.

A
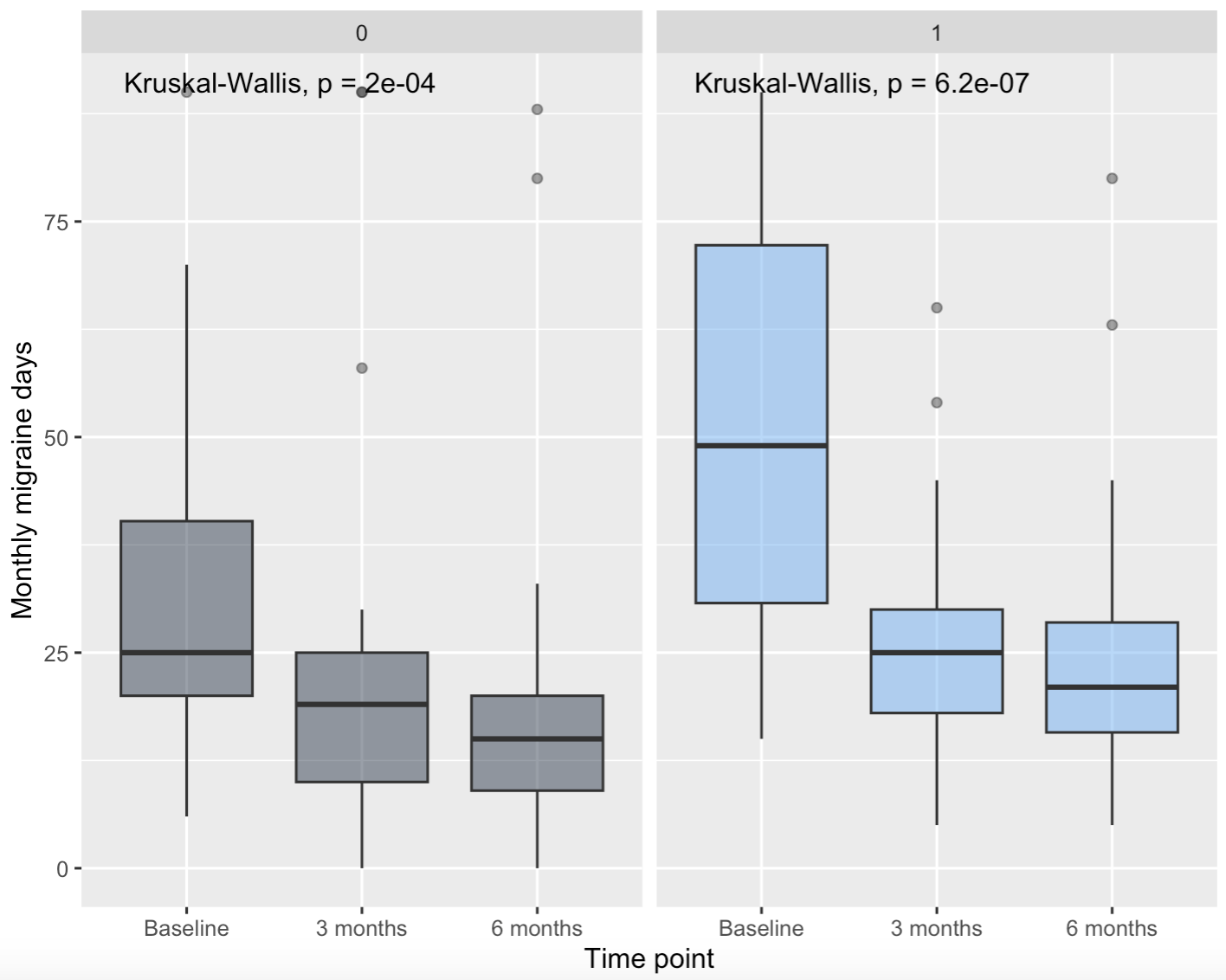
 B
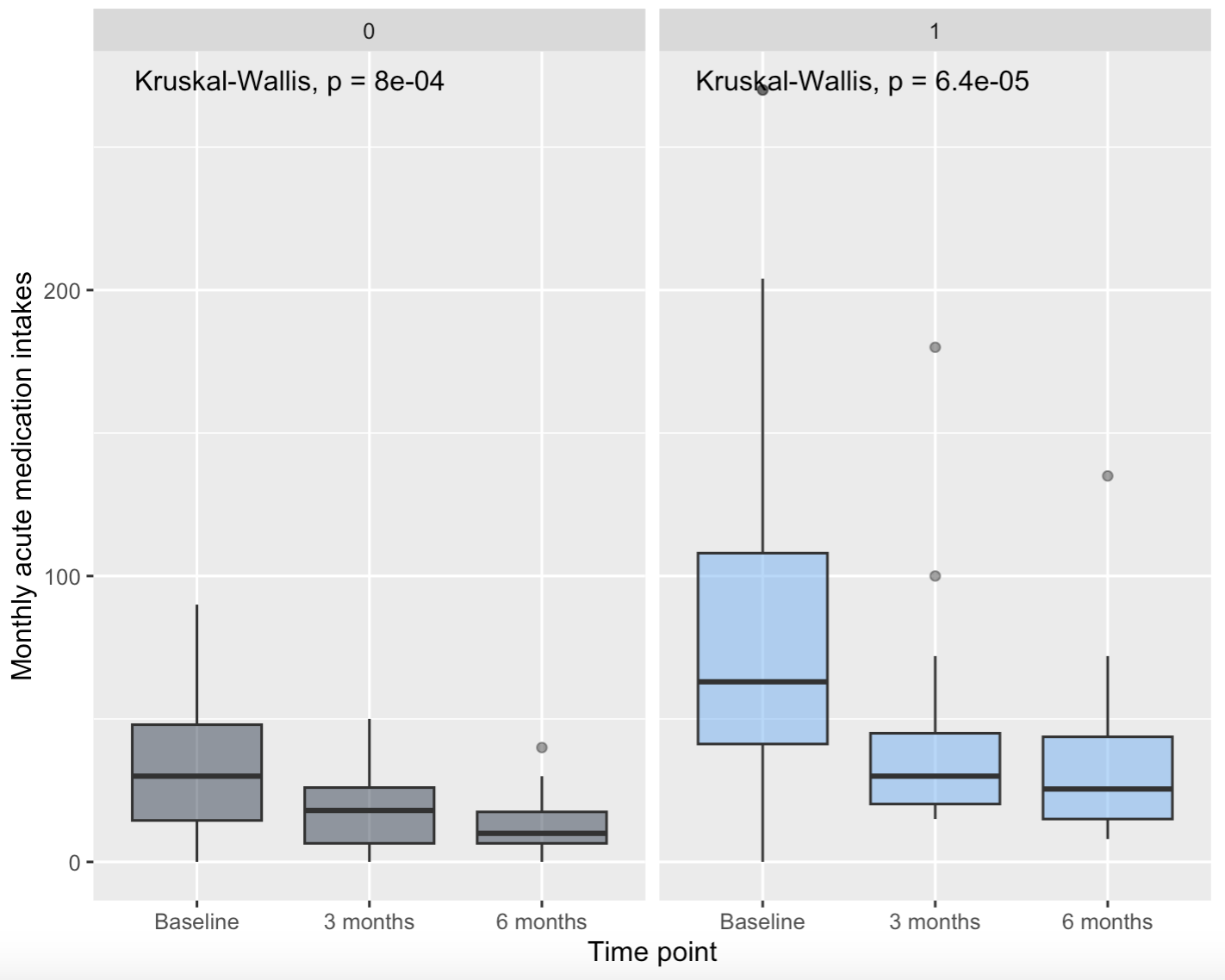


C
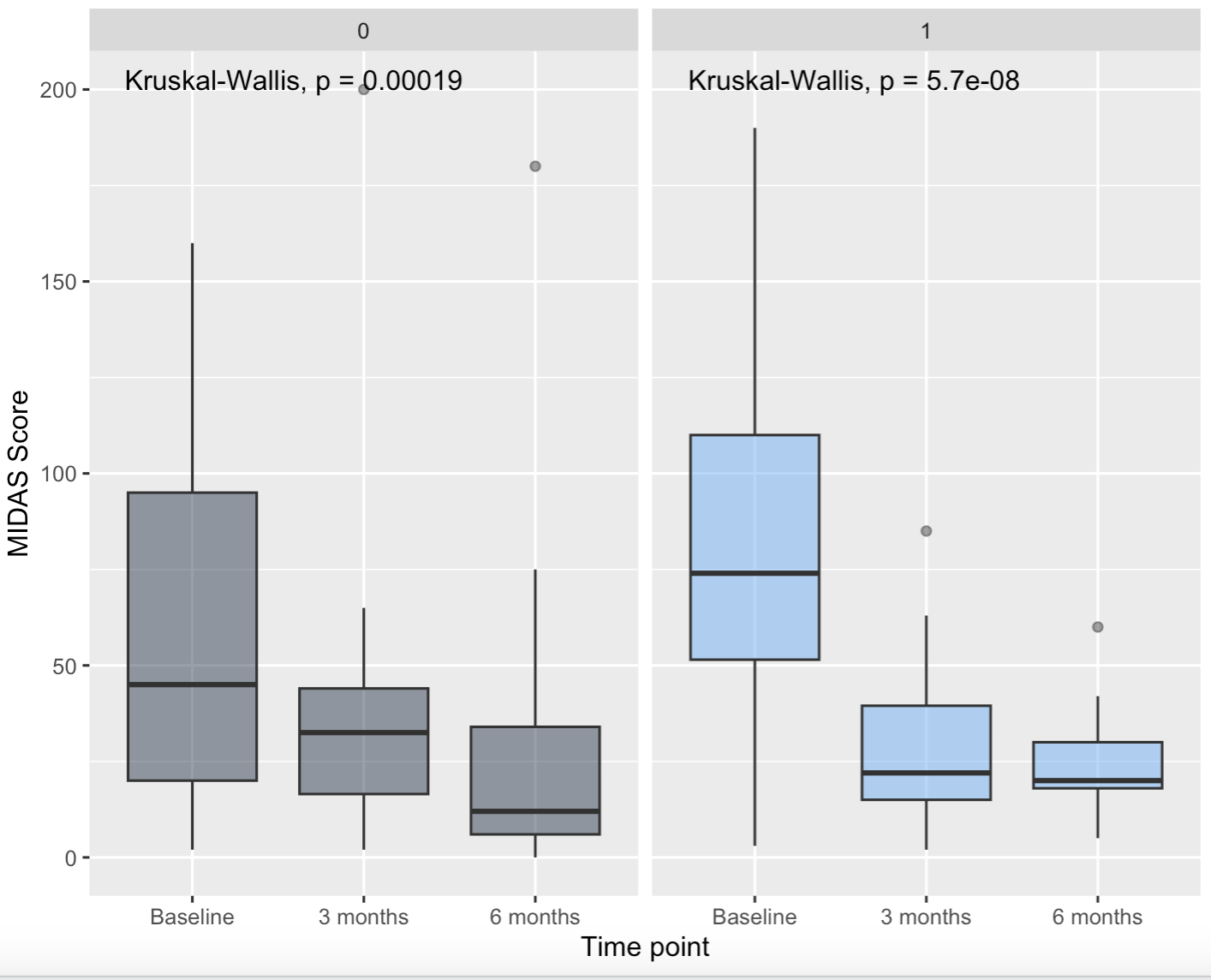
 D
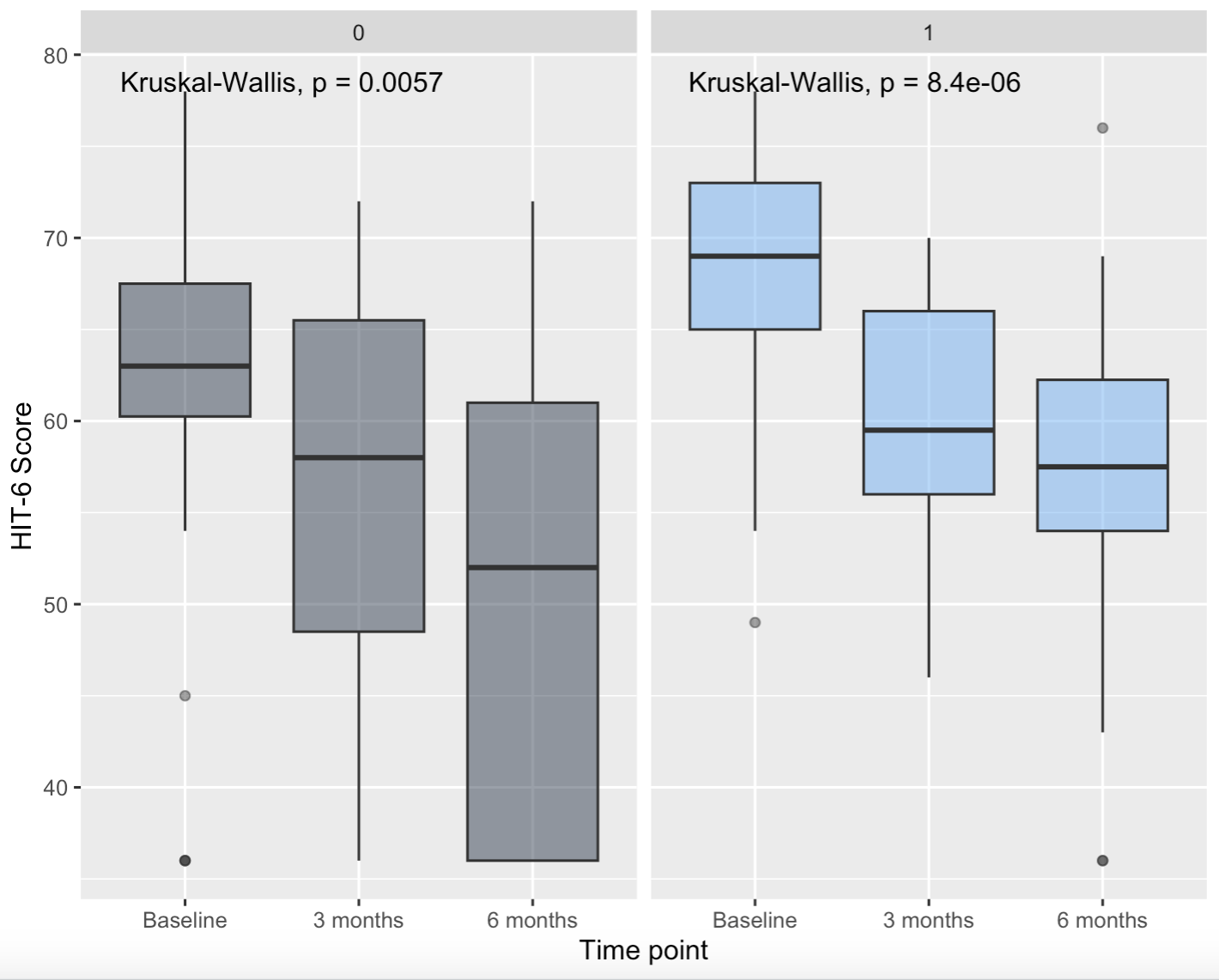

Supplement: Supplementary file 1 — Supplementary file1 (DOCX 397 KB) [file 10072_2024_7380_MOESM1_ESM.docx]
